# Rapid Switch from Intra-Aortic Balloon Pumping to Percutaneous Cardiopulmonary Support Using Perclose ProGlide

**DOI:** 10.1155/2015/407059

**Published:** 2015-12-09

**Authors:** Kenichi Sakakura, Yusuke Adachi, Yousuke Taniguchi, Hiroshi Wada, Shin-ichi Momomura, Hideo Fujita

**Affiliations:** Division of Cardiovascular Medicine, Saitama Medical Center, Jichi Medical University, 1-847 Amanuma, Omiya, Saitama 330-8503, Japan

## Abstract

We present a case of a patient who needed rapid switch from intra-aortic balloon pumping (IABP) to percutaneous cardiopulmonary support (PCPS)/venoarterial extracorporeal membrane oxygenation. It is difficult to switch from IABP to PCPS, because 0.035-inch guidewires cannot pass the IABP guidewire lumen (0.025-inch compatible), and the IABP sheath needs to be removed together with the IABP catheter. First, a 0.025-inch guidewire was inserted into the IABP wire lumen, and then the IABP catheter together with the 8 Fr IABP sheath was removed, leaving the 0.025-inch guidewire in place. We used the Perclose ProGlide for safe and rapid exchange of the 0.025-inch guidewire for a 0.035-inch guidewire. This allowed insertion of a PCPS cannula and the prompt initiation of PCPS.

## 1. Introduction

Intra-aortic balloon pumping (IABP) plays a crucial role during cardiogenic shock. However, some catastrophic situations require the use of more aggressive support such as percutaneous cardiopulmonary support (PCPS)/venoarterial extracorporeal membrane oxygenation. It is not difficult to add a PCPS cannula as long as another femoral artery is available. However, it is difficult to exchange an IABP catheter for a PCPS cannula in the same femoral artery, because a 0.035-inch guidewire cannot pass the IABP guidewire lumen (0.025-inch size). Therefore, the IABP catheter together with the 8 Fr IABP sheath needs to be removed over a 0.025-inch guidewire, and then the 0.025-inch guidewire needs to be exchanged for a 0.035-inch guidewire to introduce a PCPS cannula. It is very difficult to exchange a 0.025-inch guidewire for a 0.035-inch guidewire safely without a sheath. Also, an 8 Fr sheath compatible with a 0.025-inch guidewire is usually not available. We present a case of a patient who needed rapid switch from IABP to PCPS. We used the Perclose ProGlide (Abbott Vascular, Abbott Park, IL, USA) for safe and rapid switch from a 0.025-inch guidewire to a 0.035-inch guidewire.

## 2. Case Report

**Figure 1:**
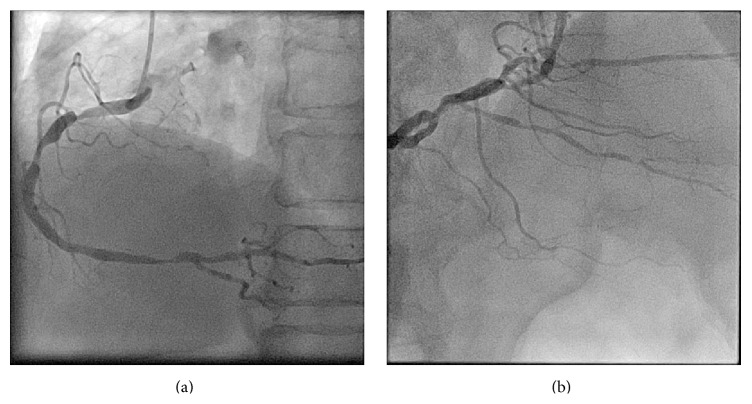
(a) Left anterior oblique view of the right coronary artery. There are three tandem stenoses. (b) Cranial view of the right coronary artery. There are several stenoses in the posterior descending branch and atrioventricular branch.

**Figure 2:**
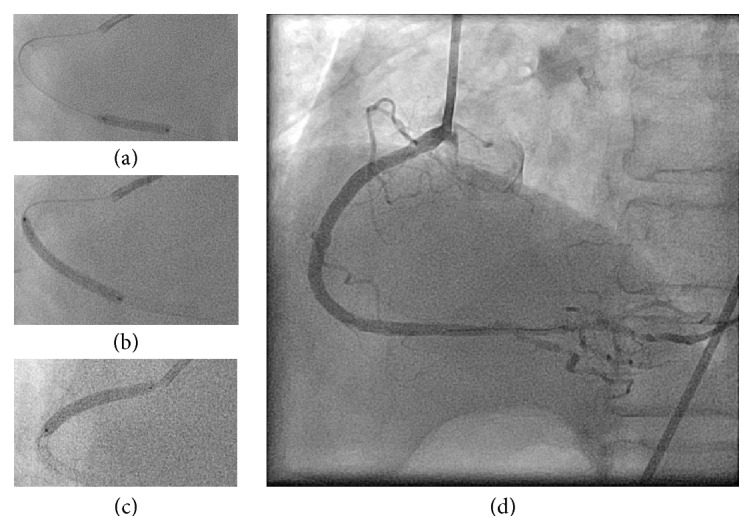
(a) A 3.0 × 18 mm everolimus-eluting stent was deployed in the distal segment of the right coronary artery. (b) A 3.0 × 38 mm everolimus-eluting stent was deployed in the middle segment of the right coronary artery. (c) A 3.5 × 38 mm everolimus-eluting stent was deployed in the proximal segment of the right coronary artery.

A 78-year-old man who had chest discomfort on exertion was referred to our medical center for coronary artery disease. He had diabetes mellitus, hypertension, and dyslipidemia as coronary risk factors; stress scintigraphy at the previous hospital revealed inferior ischemia. Coronary angiography revealed three tandem stenoses in the right coronary artery (RCA) (Figures 1(a) and 1(b)). Since the patient had been treated with dual antiplatelet therapy (aspirin and clopidogrel), we performed ad hoc percutaneous coronary intervention (PCI) of the RCA. A 7 Fr, AL1.0ST SH guiding catheter (Mach 1, Boston Scientific, Natick, MA, USA) was inserted via the right femoral artery. Three everolimus-eluting stents (3.0 × 18 mm, 3.0 × 38 mm, and 3.5 × 38 mm) were successfully deployed (Figure 2). The initial activated coagulation time (ACT) was 333 seconds, while the final ACT was 239 seconds. Protamine sulfate (10 mg) was injected intravenously, and then the 7 Fr sheath was removed from the right femoral artery. After achieving hemostasis by manual compression, the patient was moved from the catheter laboratory to a general ward.

**Figure 3:**
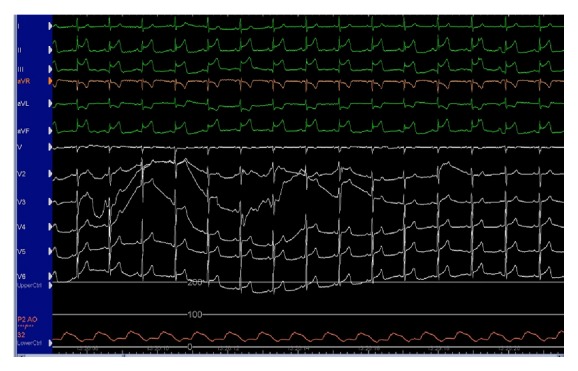
The electrocardiogram showed ST-segment elevation in leads II, III, and aVF. Aortic blood pressure with intra-aortic balloon pumping was <50 mmHg.

**Figure 4:**
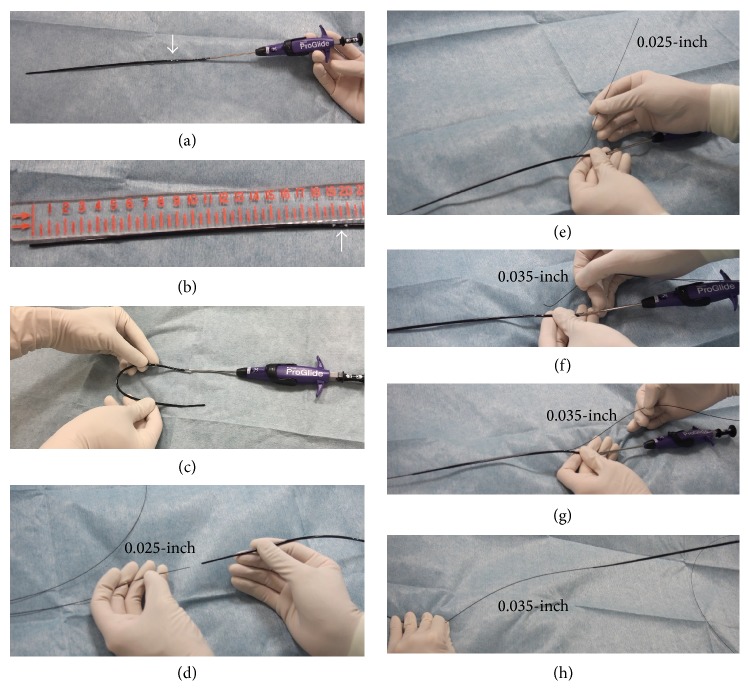
The ex vivo procedure for guidewire exchange. (a) Perclose ProGlide (Abbott vascular, Abbott Park, Illinois, USA). A white arrow shows the position of the exit port. (b) The length between the tip and exit port (white arrow) of the Perclose ProGlide is approximately 19.5 cm. (c) The rubber part of the Perclose ProGlide is flexible. (d) A 0.025-inch guidewire is inserted via the tip of the Perclose ProGlide. (e) A 0.025-inch guidewire is pulled out from the exit port of the Perclose ProGlide. (f) A 0.035-inch guidewire is inserted via the exit port of the Perclose ProGlide. (g) A 0.035-inch guidewire is advanced and pulled out from the tip of the Perclose ProGlide. (h) The Perclose ProGlide is pulled out, and the 0.035-inch guidewire is left in place. This completes the switch from a 0.025-inch guidewire to a 0.035-inch guidewire.

**Figure 5:**
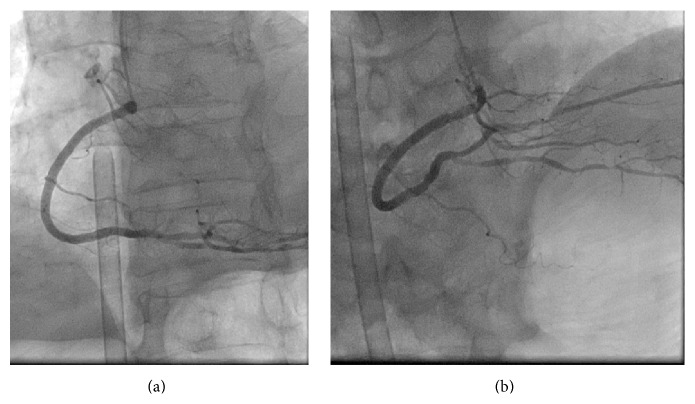
(a) Left anterior oblique view of the right coronary artery following insertion of the percutaneous cardiopulmonary support (PCPS) cannula. (b) Cranial view of the right coronary artery following PCPS cannula insertion.

One hour later, the patient returned to the catheter laboratory in shock. Although the patient was conscious, his systolic blood pressure was <50 mmHg. Furthermore, the electrocardiogram showed ST-segment elevation in leads II, III, and aVF. Therefore, we suspected that he had cardiogenic shock due to acute stent thrombosis. We inserted an 8 Fr IABP catheter (TOKAI 8 Fr IABP 40 cc L, TOKAI Medical Products, Kasugai, Japan) via the left femoral artery. At the same time, we noticed swelling of the right lower abdomen. We realized that he also had hypovolemic shock due to retroperitoneal or abdominal bleeding. We compressed the right lower abdomen manually, and normal saline and 5% albumin were rapidly injected to restore intravascular volume. Despite these measures, the patient remained in shock, and the ST elevation was sustained (Figure 3). Since we recognized that he had hypovolemic as well as cardiogenic shock, we decided to use PCPS as a life-saving intervention. Since we could not use the right femoral artery, we had to exchange the IABP catheter for a PCPS cannula via the left femoral artery. First, we inserted a 0.025-inch guidewire (220 cm) into the IABP catheter lumen and then removed the IABP catheter together with the 8 Fr IABP sheath and left the 0.025-inch guidewire in place. We then inserted a Perclose ProGlide via the 0.025-inch guidewire, removed the 0.025-inch guidewire, and left the Perclose ProGlide in place. We next inserted a 0.035-inch guidewire from the exit port of the Perclose ProGlide, removed the Perclose ProGlide, and left the 0.035-inch guidewire in place (these procedures are illustrated ex vivo in Figure 4). Since the PCPS cannula was smoothly inserted via the 0.035-inch guidewire, we started PCPS promptly. Following PCPS support, his blood pressure recovered and the ST elevation spontaneously resolved. We performed coronary angiography to check the patency of the deployed stents via the right brachial artery and confirmed that all stents were patent (Figure 5). Since there were several stenoses in the distal segments of the RCA, ischemia in the RCA territory might have been induced by insufficient coronary perfusion due to hypovolemia [1].

**Figure 6:**
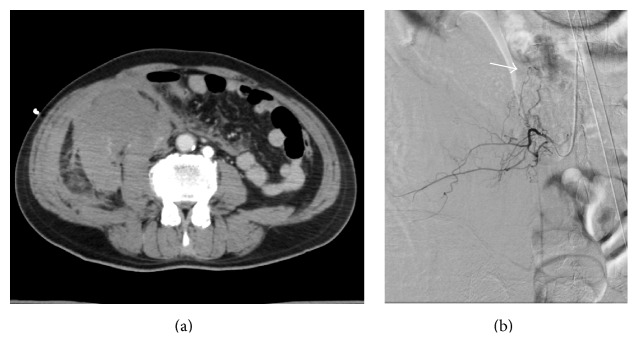
(a) Computed tomography of the abdomen revealed a large hematoma in the right abdomen. (b) Selective angiography revealed bleeding (arrow) from the right lumbar artery.

We performed abdominal computed tomography and abdominal angiography to find the source of the hemorrhage. Radiologists confirmed hemorrhage of the right lumber artery (Figure 6) and performed transcatheter arterial embolization. Since this patient was on dual antiplatelet therapy and there was substantial anticoagulation due to heparin for PCI (initial ACT, 333 seconds), spontaneous lumber artery hemorrhage was the most likely diagnosis [2, 3]. Furthermore, since we used the modified J-type guidewire (SUWANEXCEL guidewire, AuBEX, Tokyo, Japan) under fluoroscopic guidance, it is unlikely that the guidewire perforated the lumber artery. The patient was ambulatory and discharged on day 16 following PCI.

## 3. Discussion

The Perclose ProGlide, which is usually used for femoral artery closure after PCI [4], was very useful in our case for switch from a 0.025-inch guidewire to a 0.035-inch guidewire. Generally, switch from a 0.025-inch guidewire to a 0.035-inch guidewire is very easy if there is a sheath. However, since the IABP catheter is usually removed together with the 8 Fr IABP sheath, it is not easy to switch from a 0.025-inch guidewire to a 0.035-inch guidewire. Furthermore, it is very difficult to puncture the same femoral artery again if guidewire exchange fails, because of the large hole (8 Fr) that must be compressed manually. Since there is no commercially available 8 Fr sheath compatible with a 0.025-inch guidewire, we had to find a method of guidewire exchange.

Although we could not puncture the contralateral femoral artery due to hematoma, it is better to insert a PCPS cannula from the contralateral femoral artery without removal of the IABP, especially in patients with severely reduced ejection fraction [5]. However, it may be difficult to puncture the femoral artery correctly for insertion of a PCPS cannula in patients with cardiogenic shock. Our method does not need an additional puncture, which may reduce the total procedure time in patients with cardiogenic shock. Furthermore, the combination of transfemoral PCI with transfemoral IABP is common during high-risk PCI such as unprotected left main stenting [6]. We would need to stop transfemoral PCI to secure the femoral artery for PCPS, if we could not exchange an IABP catheter for a PCPS cannula.

The Perclose ProGlide has several advantages for guidewire exchange. First, the length from the tip hole to the exit port is enough long (approximately 19.5 cm) (Figure 4(b)) to ensure that the tip of device is beyond the femoral artery (it is likely in the common iliac artery), even when the patient's subcutaneous tissue is thick (obesity). Second, the tip of device is soft and flexible (Figure 4(c)), which allows the device to enter the femoral artery along with a 0.025-inch guidewire. It is usually difficult and dangerous to insert 0.035-inch guidewire compatible devices such as a conventional 8 Fr sheath over a 0.025-inch guidewire, because of the mismatch between the insufficient backup force of the 0.025-inch guidewire and the stiffness of 0.035-inch guidewire compatible devices. Finally, a Perclose ProGlide is a 6–8 Fr compatible device. Therefore, continuous bleeding from the puncture site is less likely when a Perclose ProGlide is inserted following the removal of an IABP 8 Fr sheath.

Although the Perclose ProGlide for guidewire exchange is one of the available solutions for switching from IABP to PCPS, other options are available. For example, a specific IABP catheter (MAQUET, Fairfield, NJ, USA) can be removed separately from the 8 Fr IABP sheath, thus allowing a 0.035-inch guidewire to be inserted directly via the IABP sheath. With this system, the use of a Perclose ProGlide for guidewire exchange would not be necessary; however, preparation for switching from IABP to PCPS should be done in advance in each catheter laboratory, because failure to exchange could be catastrophic in an emergency situation. This is the first case report that suggests the novel utility of a Perclose ProGlide for guidewire exchange in a life-threatening situation.

## Abbreviations

IABP: Intra-aortic balloon pumping
PCPS: Percutaneous cardiopulmonary support
RCA: Right coronary artery
PCI: Percutaneous coronary intervention
ACT: Activated coagulation time.
